# Diagnosis and Thrombolytic Management of Massive Intraoperative Pulmonary Embolism Guided by Point of Care Transthoracic Echocardiography

**DOI:** 10.1155/2018/8709026

**Published:** 2018-03-04

**Authors:** Roman Dudaryk, Julio Benitez Lopez, Jack Louro

**Affiliations:** Department of Anesthesiology, Perioperative Medicine, and Pain Management, University of Miami Miller School of Medicine, Miami, FL, USA

## Abstract

Perioperative pulmonary embolism can go undetected until the sudden onset of cardiopulmonary collapse. Point of care echocardiography in such setting can narrow the differential diagnosis of precipitous instability and facilitate tailored, rather than empiric, therapy in the event of a massive pulmonary embolism. We describe the diagnosis and successful multidisciplinary management of intraoperative massive pulmonary embolism aided by both transthoracic and transesophageal echocardiography. Key aspects regarding the classification and treatment of pulmonary embolism are subsequently reviewed.

## 1. Introduction

Perioperative pulmonary embolism (PE) presents a diagnostic challenge and confers a high risk of mortality due to limited treatment options. Despite advances in imaging modalities and clinical management, overall mortality remains high at approximately 15% [1–3]. For patients with massive pulmonary embolism and cardiac arrest, mortality exceeds 50% [2, 4]. Patients undergoing surgery often have underlying risk factors for thrombosis and embolism, such as obesity, smoking, malignancy, or traumatic injury. Furthermore, the inflammatory response to surgery leads to a prothrombotic state, which further increases the risk in conjunction with postoperative hospitalization, central venous catheterization, and immobilization. These factors account for the fivefold increased incidence of perioperative PE [5].

PE is frequently first suspected following the development of nonspecific signs, which can include hemodynamic instability, hypoxemia, increased alveolar dead space, and electrocardiogram (ECG) changes. However, intraoperatively these findings can be subtle and masked, and the first sign of PE may be sudden cardiopulmonary collapse. Furthermore, typical diagnostic modalities are intended for the hospital ward or emergency department and are impractical in the intraoperative setting. Transesophageal echocardiography (TEE) has long been recommended to aid in diagnosis of pathologies responsible for persistent hemodynamic compromise, but recently point of care transthoracic echocardiography (TTE) has emerged as a powerful and noninvasive diagnostic tool in perioperative medicine [6]. Use of TTE is very common in the ICU settings but less so in the operating room, which historically was a domain of TEE. Therefore, the use of TTE which currently has greater availability and is less invasive in the operating room for diagnosis and monitoring of the response to hemodynamic intervention in patients with suspected acute cor pulmonale is underappreciated by anesthesiologists. Patients with significant PE can benefit from surgical or pharmacological thrombolysis, and thus rapid diagnosis and treatment are crucial to improve outcomes [3]. Herein, we describe the utility of structured intraoperative TTE and TEE examinations to aid in the diagnosis and management of massive PE. The patient provided signed and witnessed written consent to publish this deidentified case report.

## 2. Case Description

**Figure 1:**
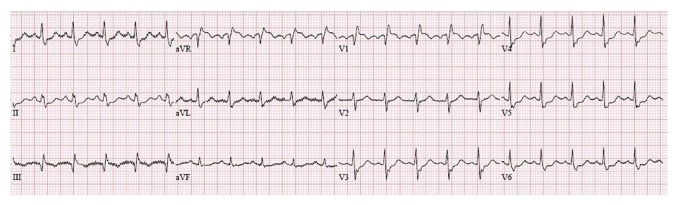
Intraoperative 12 lead electrocardiogram demonstrating signs of acute cor pulmonale: S waves in lead 1 and Q waves with the suggestion of T wave inversion in lead 3.

**Figure 2:**
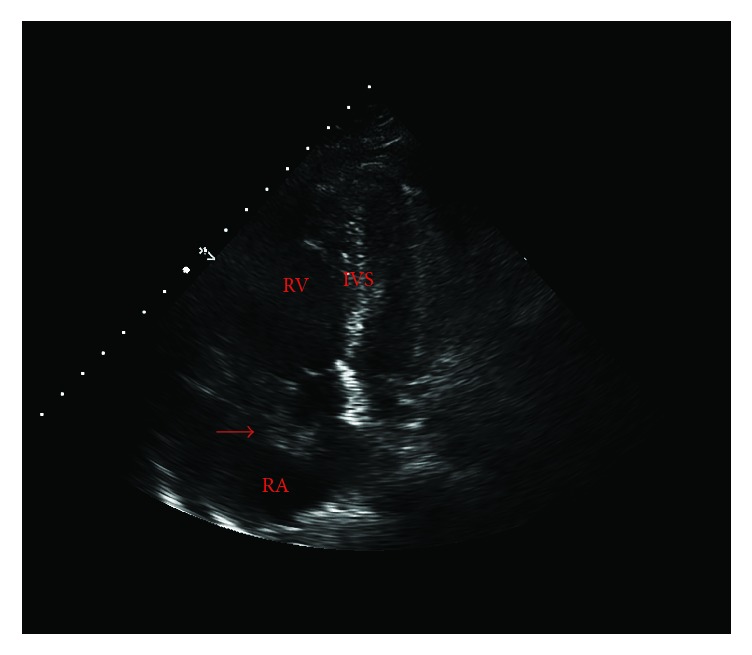
Intraoperative transthoracic echocardiography, 4 chamber apical view showing a right atrial thrombus (arrow), right ventricular dilatation, right atrial dilatation, and septal bowing. RV, right ventricle; RA, right atrium; IVS, interventricular septum; LA, left atrium; LV, left ventricle.

A 59-year-old male bicyclist with no past medical history presented to the trauma resuscitation unit at the Ryder Trauma Center in Miami, FL, after being struck by a motor vehicle. He sustained a comminuted left subtrochanteric femur fracture and was taken to the operating room for an open reduction and fixation. Following a rapid sequence induction of general anesthesia with propofol and succinylcholine, the patient was readily intubated via direct laryngoscopy. Approximately 5 minutes after induction, initial skin preparation and positioning were begun by the orthopedic surgery team. Upon initial manipulation of the left lower extremity, the anesthesiologist noticed a precipitous drop in the end-tidal carbon dioxide (EtCO2), heart rate greater than 120 bpm, and systolic blood pressure less than 90 mmHg. The surgical team was informed, and manipulation of the lower extremities was suspended. The patient was immediately treated with phenylephrine and crystalloid fluid boluses. The patient had oxygen desaturation to the low 90s, which responded to an increase in fractional inspired (FiO2), and a transient drop in EtCO2 of greater than 15 mmHg, which were concerning for a sudden increase in dead space ventilation. A 12-lead EKG showed signs of right heart strain, including an S wave in lead 1 and Q waves with T wave inversion in lead 3 (Figure 1). Given this constellation of findings in conjunction with hemodynamic instability, preparations were begun to facilitate a TEE examination. In the interim, a structured TTE examination according to the FATE protocol (Focused Assessed Transthoracic Echocardiography) was immediately performed [7]. The parasternal long axis view was remarkable for massive dilatation and hypokinesia of the right ventricle (RV). In the parasternal short axis, flattening of the intraventricular septum (D-shaped) and paradoxical septal motion were evident. The flattening of interventricular septum at end-systole suggested RV pressure-overload, probably due to distal pulmonary artery obstruction, and enabled us to make differential diagnosis from RV volume overload, as the reason for hemodynamic deterioration. The apical four-chamber view revealed a mobile mass in the right atrium (RA) suspicious for a thrombus (Figure 2, Supplemental Video 1). As this constellation of findings was pathognomonic for massive PE, the surgical procedure was halted.

**Figure 3:**
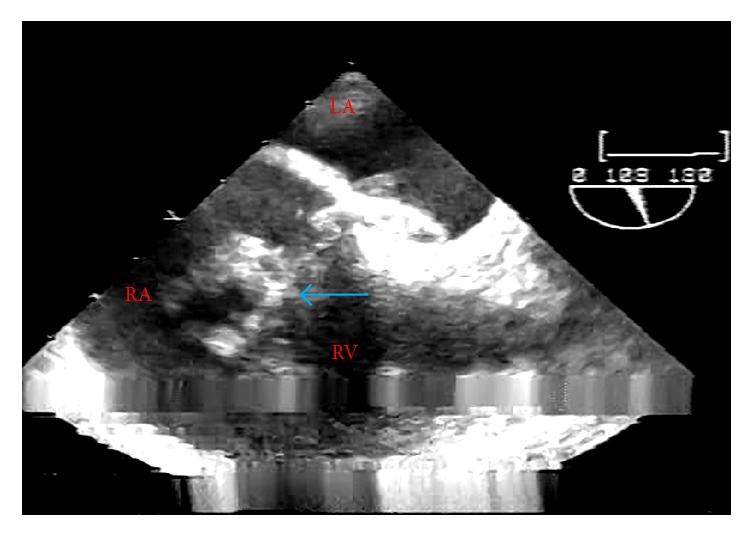
Initial intraoperative transesophageal echocardiography; midesophageal 4-chamber view. A large right atrial thrombus was visualized (blue arrow). LA, left atrium; RA, right atrium; RV, right ventricle.

**Figure 4:**
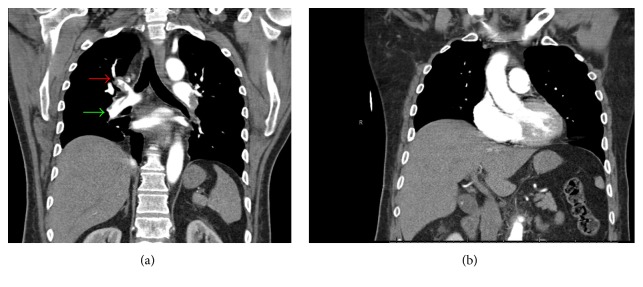
Perioperative chest computed tomography angiography; coronal reconstructions. (a) Filling defects were noted in right upper (red arrow) and lower lobar (green arrow) pulmonary arteries. (b) Right ventricular dilatation.

A TEE was then performed by the anesthesia team, which facilitated more detailed evaluation of the RA. In the midesophageal 4-chamber view, a patent foramen ovale and associated RA thrombus were evident (Figure 3, Supplemental Video 2). Cardiothoracic surgery, interventional radiology, and intensivists from the Trauma Intensive Care Unit (ICU) were consulted intraoperatively. A repeat TEE examination after their arrival failed to demonstrate either the previously seen RA clot or any thrombus within the visualized portions of the pulmonary artery (PA). As such, probable migration was suspected. At this point in time, the contractility of the RV on TEE was noted to be improved with inotropic and vasopressor support despite dilatation and pressure overload. This allowed us to make the decision regarding the transport of the patient to the radiography suite for definitive treatment. No changes in hemodynamics or oxygenation were noted at this time, aside from occasional phenylephrine doses. Following a multidisciplinary discussion, the patient was anticoagulated with a heparin infusion, and a computed tomography (CT) angiogram of the chest was obtained to further evaluate the thrombus and guide the course of treatment. At this juncture, the patient was hemodynamically stable and thus deemed suitable for transport. He was brought intubated and sedated to the radiology department by the anesthesia and surgical team. CT angiography revealed RV dilatation with a large distal right PA thrombus extending into the upper and lower lobar arteries, along with a left upper and lower lobar artery thrombus (Figure 4). Evaluation by cardiothoracic surgery concluded that the thrombi were too distal as they were no longer seen on the repeat TEE exam and only detectable on CT for effective surgical embolectomy. As the patient was still requiring vasopressor and inotropic support with RV dilatation evident on echocardiography, the next step in management required some form of thrombolysis. Interventional radiology deemed him a good candidate for catheter-directed ultrasound accelerated thrombolysis. Ultrasound accelerated thrombolysis catheters measuring 24 and 12 cm were placed into the right and left PA, respectively. The patient was subsequently admitted to the ICU for further monitoring and optimization. Tissue plasminogen activator (tPA) was infused through each catheter at 0.75 mg/hr with a normal saline carrier at 35 ml/hr.

On postoperative day (POD) 2, a formal TTE was performed that demonstrated improved RV function with only slightly increased RV systolic pressures. The patient was extubated on POD 4 and later underwent an uneventful definitive fixation of fractures on hospital day 11. He was bridged to warfarin and discharged to a rehabilitation facility on hospital day 22.

## 3. Discussion

Rapid and reliable diagnosis of massive PE is critical to identify patients who will benefit from anticoagulation, surgical embolectomy, pharmacological thrombolysis, or catheter directed therapies. As commitment to these treatment options confers a high risk of complications, stratification schemas have been developed to identify patients at a high risk for mortality for whom the benefits of treatment are likely to outweigh the risks. As such, the current classification of PE by the American Heart Association (AHA) as massive, submassive, or low-risk has moved away from the historical model of categorization by degree of clot burden and instead focuses on the presence or absence of symptoms associated with adverse outcomes [8]. The AHA defines massive PE as an acute event coupled with sustained hypotension (systolic blood pressure less than 90 mmHg for more than 15 minutes or requiring inotropic support), pulselessness, or bradycardia in the absence of other causes. Submassive PE, by definition, is not accompanied by sustained hypotension but does involve signs of RV dysfunction or myocardial necrosis as evidenced by certain echocardiographic findings, ECG features, laboratory studies, and clinical scores. These patients are still at a heightened risk for adverse events and require emergent interventions intended to decrease clot burden and improve mortality [8]. Low-risk PE describes patients with the best prognosis, typically those with normal hemodynamics, normal biomarkers, and no signs of RV dysfunction.

Both TEE and TTE have been increasingly utilized to aid in the rapid diagnosis of PE, evaluation of RV strain, and detection of cardiac sequelae. In one case series, the sensitivity of TEE for the detection of massive central PE was found to be about 80% with 100% specificity [9]. However, more distal thromboembolic burden is difficult to visualize via echocardiography, as evidenced by an intraoperative case series wherein PE was directly visualized in only 26% of cases [10]. However, TEE can often reveal extrapulmonary venous or intracardiac emboli, which can easily influence surgical planning [11, 12]. As such, direct visualization of thrombus is specific but not sensitive. Indirect evidence of pulmonary embolism, including RV dysfunction and leftward interatrial septal bowing, has been found in over 96% of patients, and acute tricuspid regurgitation is present in more than 50% of patients undergoing emergent embolectomy [10].

Historically, assessment of RV function by echocardiography is challenging, due its complex geometry, lack of standardized imaging planes, and numerous assumptions required [13, 14]. As such, there is no one single dimension, sign, or parameter that allows for reliable assessment of RV function [14]. Increased evidence of the clinical implications of RV dysfunction and associated outcomes has led to the creation of guidelines and recommendations to standardize RV assessment [13]. In brief, echocardiographic evaluation RV function includes both qualitative assessment and evaluation of quantitative parameters. Patterns describing RV ejection have been shown to be highly specific, including “McConnell's sign” (e.g., RV free wall hypokinesis with preserved contraction of the apex) and the “60-60 sign” (e.g., a tricuspid regurgitation gradient less than 60 mmHg with pulmonary flow acceleration less than 60 milliseconds) [14]. Additional recommended RV evaluation parameters include RV, right atrial, and inferior vena cava dilation, as well as markers of RV systolic function including fractional area change, tricuspid annular plane systolic excursion (TAPSE), RV index of myocardial performance (RIMP), and Doppler estimation of PA pressures [13]. All of these signs and indices must ultimately be combined and interpreted in the context of subjective and clinical assessment.

The treatment of massive PE in the perioperative period typically involves a multidisciplinary discussion to facilitate risk to benefit ratio analysis. Numerous reperfusion therapies are available and include systemic fibrinolysis, catheter directed therapies (fibrinolysis or thrombectomy), and surgical embolectomy. The lack of current consensus, or evidence-driven guidelines in the perioperative period, makes treatment decisions challenging. Systemic anticoagulation and thrombolysis have been used successfully for surgical patients [15–17]. However, pharmacologic treatment may be contraindicated in the setting of recent surgery given a potentially heightened risk of major hemorrhage. Surgical embolectomy is recommended in high-risk PE patients with a contraindication to, or failure of, systemic thrombolysis [14]. Recent evidence suggests low mortality rates in patients undergoing early surgical embolectomy with hemodynamically stable, high-risk PE and echocardiographic signs of RV dysfunction [18, 19]. The signs of right ventricular dysfunction were evident in our patient on TEE. However, the emboli were too distal for effective surgical treatment as they were noted to migrate from the RA on the second echocardiographic evaluation. In the case of this patient, systemic fibrinolysis was deemed to confer a high risk for hemorrhage, but more aggressive thrombolysis was deemed necessary as RV dilatation and dysfunction were clear on TEE. If the patient had normal RV function after supportive therapy and initiation of heparin on echocardiography, management would have involved anticoagulation with heparin in the ICU as opposed to attempts at thrombolysis. Echocardiography was able to help guide the management approach by revealing the right ventricular dysfunction.

Ultrasound accelerated fibrinolysis catheters are a novel treatment alternative and increasingly available amongst experienced centers. This technology was recently approved by the FDA for the treatment of PE. These catheters deliver low power, high frequency ultrasound waves that hypothetically expose plasminogen receptors sites on the fibrin molecules, which enhances the efficacy of tPA [20]. They have been shown in a small multicenter randomized controlled trial to be more effective than heparin alone in reducing RV strain without an increase in bleeding complications [21].

In the case of this patient, intraoperative TTE and TEE lead to the rapid diagnosis of PE by direct visualization and identification of RV dysfunction, which facilitated immediate multidisciplinary discussion and treatment. Intraoperative diagnosis of the PE led to immediate cessation of manipulation, potentially avoiding further thromboembolic burden from the lower extremity. Due to the changes observed on TEE, systemic anticoagulation was initiated intraoperatively and CT chest angiography was obtained to assess for residual embolism. A multidisciplinary discussion of risks/benefits, PE distribution, and patient condition, along with center-specific resources and experience, facilitated effective treatment. Ultrasound accelerated catheter directed thrombolysis with systemic anticoagulation resulted in normalization of RV function and discharge to rehabilitation without complication.

## Supplementary Materials

Supplementary Materials Supplemental Video 1: intraoperative transthoracic echocardiography and 4-chamber apical view showing a right atrial thrombus, right ventricular dilatation, right atrial dilatation, septal bowing, and paradoxical septal motion. Supplemental Video 2: intraoperative transesophageal echocardiography, modified midesophageal (ME) 4-chamber view transitioning into ME aortic valve short axis, and ME bicaval view. Large right atrial thrombus seen as well as right atrial, right ventricular dilatation with paradoxical septal motion.

## References

[1] Goldhaber S. Z., Visani L., de Rosa M. (1999). Acute pulmonary embolism: clinical outcomes in the International Cooperative Pulmonary Embolism Registry (ICOPER). *The Lancet*.

[2] Kucher N., Rossi E., De Rosa M., Goldhaber S. Z. (2006). Massive pulmonary embolism. *Circulation*.

[3] Wood K. E. (2002). Major pulmonary embolism: review of a pathophysiologic approach to the golden hour of hemodynamically significant pulmonary embolism. *CHEST*.

[4] Kürkciyan I., Meron G., Sterz F. (2000). Pulmonary embolism as a cause of cardiac arrest: presentation and outcome. *Archives of Internal Medicine*.

[5] Desciak M. C., Martin D. E. (2011). Perioperative pulmonary embolism: diagnosis and anesthetic management. *Journal of Clinical Anesthesia*.

[6] Thys D. M., Brooker R. F., Cahalan M. K. (2010). American Society of A, Society of Cardiovascular Anesthesiologists Task Force on Transesophageal E. Practice guidelines for perioperative transesophageal echocardiography. An updated report by the American Society of Anesthesiologists and the Society of Cardiovascular Anesthesiologists Task Force on Transesophageal Echocardiography. *Anesthesiology*.

[7] Jensen M. B., Sloth E., Larsen K. M., Schmidt M. B. (2004). Transthoracic echocardiography for cardiopulmonary monitoring in intensive care. *European Journal of Anaesthesiology*.

[8] Jaff M. R., McMurtry M. S., Archer S. L. (2011). Management of massive and submassive pulmonary embolism, iliofemoral deep vein thrombosis, and chronic thromboembolic pulmonary hypertension: a scientific statement from the american heart association. *Circulation*.

[9] Pruszczyk P., Torbicki A., Pacho R. (1997). Noninvasive diagnosis of suspected severe pulmonary embolism: Transesophageal echocardiography vs spiral CT. *CHEST*.

[10] Rosenberger P., Shernan S. K., Body S. C., Eltzschig H. K. (2004). Utility of intraoperative transesophageal echocardiography for diagnosis of pulmonary embolism. *Anesthesia and Analgesia*.

[11] Langeron O., Goarin J. P., Pansard J. L., Riou B., Viars P. (1992). Massive intraoperative pulmonary embolism: diagnosis with transesophageal two-dimensional echocardiography. *Anesthesia and Analgesia*.

[12] Rosenberger P., Shernan S. K., Weissmüller T. (2005). Role of intraoperative transesophageal echocardiography for diagnosing and managing pulmonary embolism in the perioperative period. *Anesthesia & Analgesia*.

[13] Rudski L. G., Lai W. W., Afilalo J. (2010). Guidelines for the echocardiographic assessment of the right heart in adults: a report from the American Society of Echocardiography endorsed by the European Association of Echocardiography, a registered branch of the European Society of Cardiology, and the Canadian Society of Echocardiography. *Journal of the American Society of Echocardiography*.

[14] Konstantinides S. V., Torbicki A., Agnelli G. (2014). 2014 ESC Guidelines on the diagnosis and management of acute pulmonary embolism: The Task Force for the Diagnosis and Management of Acute Pulmonary Embolism of the European Society of Cardiology (ESC) Endorsed by the European Respiratory Society (ERS). *European Heart Journal*.

[15] Wenk M., Pöpping D. M., Hillyard S., Albers H., Möllmann M. (2011). Intraoperative thrombolysis in a patient with cardiopulmonary arrest undergoing caesarean delivery. *Anaesth Intensive Care*.

[16] Zhang K., Zeng X., Zhu C. (2013). Successful thrombolysis in postoperative patients with acute massive pulmonary embolism. *Heart, Lung and Circulation*.

[17] Sondekoppam R. V., Kanwar M., Latha Y. S., Mandal B. (2013). High dose streptokinase for thrombolysis in the immediate postoperative period: a case report. *Middle East Journal of Anaesthesiology*.

[18] Carvalho E. M., MacEdo F. I. B., Panos A. L., Ricci M., Salerno T. A. (2010). Pulmonary embolectomy: recommendation for early surgical intervention. *Journal of Cardiac Surgery*.

[19] Leacche M., Unic D., Goldhaber S. (2005). Modern surgical treatment of massive pulmonary embolism: results in 47 consecutive patients after rapid diagnosis and aggressive surgical approach. *The Journal of Thoracic and Cardiovascular Surgery*.

[20] Francis C. W., Blinc A., Lee S., Cox C. (1995). Ultrasound accelerates transport of recombinant tissue plasminogen activator into clots. *Ultrasound in Medicine & Biology*.

[21] Kucher N., Boekstegers P., Muller O. J. (2014). Randomized, controlled trial of ultrasound-assisted catheter-directed thrombolysis for acute intermediate-risk pulmonary embolism. *Circulation*.

